# Inhibition of bone morphogenetic protein 6 receptors ameliorates Sjögren’s syndrome in mice

**DOI:** 10.1038/s41598-020-59443-z

**Published:** 2020-02-19

**Authors:** Hongen Yin, Lovika Kalra, Zhennan Lai, Maria C. Guimaro, Lauren Aber, Blake M. Warner, Drew Michael, Nan Zhang, Javier Cabrera-Perez, Arif Karim, William D. Swaim, Sandra Afione, Alexandria Voigt, Cuong Q. Nguyen, Paul B. Yu, Donald B. Bloch, John A. Chiorini

**Affiliations:** 1 AAV Biology Section, Division of Intramural Research, National Institute of Dental and Craniofacial Research, National Institutes of Health, Bethesda, MD USA; 2 Department of Pathology and Infectious Diseases, University of Florida, Gainesville, FL USA; 3 Cardiovascular Division, Brigham and Women’s Hospital, Harvard Medical School, Boston, MA USA; 4 Center for Immunology and Inflammatory Diseases and the Division of Rheumatology, Allergy, and Immunology of the Department of Medicine, Massachusetts General Hospital, Harvard Medical School, Boston, MA USA

**Keywords:** Rheumatology, Rheumatic diseases

## Abstract

Primary Sjögren’s syndrome (pSS) is a chronic autoimmune disease, with only palliative treatments available. Recent work has suggested that increased bone morphogenetic protein 6 (BMP6) expression could alter cell signaling in the salivary gland (SG) and result in the associated salivary hypofunction. We examined the prevalence of elevated BMP6 expression in a large cohort of pSS patients and tested the therapeutic efficacy of BMP signaling inhibitors in two pSS animal models. Increased BMP6 expression was found in the SGs of 54% of pSS patients, and this increased expression was correlated with low unstimulated whole saliva flow rate. In mouse models of SS, inhibition of BMP6 signaling reduced phosphorylation of SMAD1/5/8 in the mouse submandibular glands, and led to a recovery of SG function and a decrease in inflammatory markers in the mice. The recovery of SG function after inhibition of BMP6 signaling suggests cellular plasticity within the salivary gland and a possibility for therapeutic intervention that can reverse the loss of function in pSS.

## Introduction

Primary Sjögren’s syndrome (pSS) is a chronic autoimmune disease that occurs predominately in women, with a female to male ratio of 9–16:1^1,2^. Hallmarks of the disease include a loss of lacrimal and salivary gland (SG) function, the presence of lymphocytic infiltrates in SGs, and increased levels of proinflammatory cytokines and circulating autoantibodies. Patients report significant fatigue and a decreased quality of life. The etiology of pSS is unknown and treatment is limited to symptomatic care^2^.

Previous studies reported that expression of bone morphogenetic protein 6 (BMP6) is increased in the SGs of some pSS patients and that this overexpression is linked to a decrease in SG function and increased lymphocytic infiltration of the gland^3,4^. Overexpression of BMP6 in the SGs of C57BL/6 mice results in a Sjögren’s syndrome-like phenotype^3^. However, the downstream signals that are activated by BMP6 and induce SG dysfunction and autoimmunity, are still unknown. Gene therapy experiments in these mice have shown that reengineering the glandular epithelia to express aquaporin 1 (AQP1), to restore membrane water permeability, recovers some of the secretory function of the SG epithilia^4^. However, AQP1 gene therapy, does not correct all of the effects of increased expression of the BMP6 ligand that likely affects the activity of other cells in the gland, such as infiltrating lymphocytes and bone marrow mesenchymal stem cells^5,6^. Therefore, a systemic treatment that targets the fundamental signaling problem may be useful in correcting the loss of immune homeostasis associated with the autoimmunity seen in SS.

The TGF-β cytokine superfamily is a group of 33 proteins, which include bone morphogenetic protein (BMP) and TGF members. Members of this family bind to and signal through specific type I and type II transmembrane serine/threonine kinase receptor complexes. The particular ligand determines the downstream signaling, which then activates specific intracellular SMAD transcription factors^7–10^. Four type I receptors (ALK1/ACVRL1, ALK2/ACVR1, ALK3/BMPR1A and ALK6/BMPR1B) are involved in BMP signaling through phosphorylation of SMAD1/5/8. The other three type I receptors are ALK4/ACVR1B, ALK7/ACVR1C, and the TGF-β receptor type I (ALK5/TGFBR1) signal through SMAD2/3. Lastly, BMP type I receptors can form an oligomeric complex with type II receptors (BMPR2, ActRIIA, and ActRIIB).

BMP6 signaling is involved in a wide range of biological processes, including iron homeostasis, fat and bone development, and ovulation, as well as stem cell self-renewal and differentiation^11,12^. The central role of BMP signaling has led to the development of specific small-molecule kinase inhibitors. Recent work has identified the BMP signaling inhibitors LDN-212854 and LDN-193189 as highly selective for ALK2 and ALK3 receptors compared with other type I receptors^13^. Some difference in activity exists between these inhibitors with LDN-212854 exhibiting greater selectivity towards ALK2 than ALK3 compared to LDN-193189^13–15^.

In the present study, the ability of these inhibitors to block BMP6 signaling was investigated in two SS mouse models and in HSG cells and evaluated for the ability to block the expression of key proteins involved in SG function and inflammation. Treatment of HSG cells *in vitro* with LDN-212854 or LDN-193189 resulted in decreased BMP6 signaling and SMAD1/5/8 phosphorylation and led to a recovery of fluid movement across the cell membrane. Daily treatment of BMP6-overexpressing mice with LDN-193189 or C57BL/6.NOD-*Aec1Aec2* mice with either LDN-212854 or LDN-193189 restored SG function in mice with established disease. Associated with this functional increase was an increase in expression of AQP5, a protein critical for membrane water permeability in SGs^16^. Treatment with either BMP signaling inhibitor also decreased the infiltration of interferon gamma (IFN-γ) producing CD4^+^ T cells in the submandibular glands (SMGs) of C57BL/6.NOD-*Aec1Aec2* mice. Our findings suggest that the use of small molecule inhibitors of BMP6 signaling is a promising approach for the treatment of pSS.

## Methods

### Cells

HSG cells were provided by Dr. Indu Ambudkar (National Institute of Dental and Craniofacial Research [NIDCR], National Institutes of Health [NIH]), and cultured in Dulbecco’s Modified Eagle Medium (DMEM, Thermo Fisher Scientific, Waltham, MA, USA) supplemented with 10% fetal bovine serum (FBS) in a humidified incubator at 37 °C with 5% CO_2_. HSG cells, which based on short tandem repeat analysis share a common origin with Hela cells, have been used as a model to test regulatory volume decrease (RVD) as a surrogate assay for water movement across a membrane and the molecular mechanisms of secretion from exocrine tissue^4,17,18^.

### Patient selection criteria

Studies involving healthy subjects were conducted in accordance with approved National Institute of Health (NIH) guidelines. All participants provided informed consent prior to the initiation of any study procedures. Healthy volunteer samples were obtained from NIH Institutional Review Board approved protocols in the Sjögren’s Syndrome Clinic at the National Institute of Dental and Craniofacial Research (NIDCR) at the NIH in Bethesda, MD. The protocols utilized in this study are registered at ClinicalTrials.gov (NCT00001390, NCT00001852). In addition, a sequential cohort of seventy-nine deidentified female patients with pSS were selected from the Sjögren’s International Collaborative Clinical Alliance (SICCA). All patients fulfilled the 2016 American College of Rheumatology/European League Against Rheumatism (ACR/EULAR) classification criteria for pSS^19^. Their clinical manifestations are summarized in Supplemental Table 1.

### *In vitro* treatment of HSG cells with ALK2/3 inhibitors

HSG cells were plated at 4 × 10^5^ cells per well in 35-mm plates, and cultured in 2 mL of DMEM/Nutrient Mixture F-12 (DMEM/F-12) with 5% FBS. After 24 h, medium was switched to low-serum medium containing DMEM/F-12 with 0.2% FBS. After a 24 h incubation, cells were treated with the following reagents for an additional 24 h: LDN-212854 (Cat# SML0965, Sigma-Aldrich Corp., The Woodlands, TX, USA) or LDN-193189 (Cat# SML0559, Sigma-Aldrich Corp.), 10 or 60 nM; recombinant human BMP6 (Cat# 507-BP-020, R&D Systems, Minneapolis, MN, USA), 6 or 25 ng/mL; and recombinant human TGF-β1 (Cat# 240-B-002, R&D Systems), 5 ng/mL. For inhibitors studies, BMP6 was added to the medium together with LDN-212854 or LDN-193189, while TGF-β1 was added during the last 45 minutes (min) of this treatment, before cells were harvested. The resuspension medium (DMSO or H_2_O) was used as the negative control for LDN-212854 or LDN-193189 respectively.

### Western blot analysis of SMAD signaling

To analyze signaling downstream of BMP6 activation, we studied phosphorylation of SMAD proteins. For the *in vitro* studies, HSG cells were harvested after treatment and whole-cell lysates were prepared using RIPA Lysis and Extraction Buffer with Halt Protease and Phosphatase Inhibitor Cocktail (Cat# 89900 and 78441, Thermo Fisher Scientific, Waltham, MA, USA). Cells were scraped, sonicated, and then incubated on ice for 20 min. Clarified supernatants were collected from whole-cell lysates that were centrifuged at 14,000 rpm at 4 °C for 20 min. The total protein concentration of the supernatants was measured with the Quick Start Bradford Protein Assay (Bio-Rad Laboratories, Hercules, CA, USA) with bovine serum albumin (BSA) as protein standard. Forty micrograms of protein from each lysate was denatured with 4× Laemmli Sample Buffer and 2-mercaptoethanol (both Bio-Rad Laboratories) at 75 °C for 10 min prior to fractionation on a 4–12% SDS-PAGE gel (Invitrogen, Frederick, MD, USA). The proteins were then transferred to a nitrocellulose membrane using the Trans-Blot Turbo Mini Nitrocellulose Transfer Pack (Bio-Rad Laboratories) at 1.3 A and 25 V for 12 min.

The following antibodies from Cell Signaling Technology (Danvers, MA, USA) were used: anti-pSMAD1/5/8 (Cat# 9516), anti-pSMAD2/3 (Cat# 8828), and anti-SMAD2 (1:1,000; Cat# 3103). Anti-SMAD1/5/8 antibody (1:400; Cat# sc-6031-R) was purchased from Santa Cruz Biotechnology (Santa Cruz, CA, USA) were used to study SMAD phosphorylation. For anti-pSMAD1/5/8 (diluted 1:1,000) and anti-pSMAD2/3 (diluted 1:1,000 antibodies, 5% BSA in 1x Tris-Buffered Saline with 0.1% Tween 20 (TBST) was used as blocking and incubation buffer for all steps; for all other antibodies, 5% nonfat milk in TBST was used. Similar protein loading was confirmed by using peroxidase-conjugated anti-β-actin antibody (1:500,000; Cat# A3854, Sigma-Aldrich Corp.). Expression of phosphorylated and total protein was quantified by densitometry scanning and analyzed using ImageJ software (NIH, Bethesda, MD, USA). The ratios of pSMAD1/5/8 to total SMAD1/5/8 and pSMAD2/3 to total SMAD2 were calculated and used to determine changes in SMAD phosphorylation.

### *In vivo* studies of mice with Sjögren’s syndrome-like phenotype

The C57BL/6.NOD-*Aec1Aec2* mouse model of Sjögren’s syndrome-like phenotype was used to examine exocrine gland dysfunction, lymphocytic infiltration, autoantibody formation, and increased serum proinflammatory cytokines^20,21^. C57BL/6.NOD-*Aec1Aec2* mice (male and female) were bred and maintained, as described previously, at the animal facility of the Department of Pathology of the University of Florida^4,22^. To create a second murine model of pSS, a viral vector encoding BMP6 was delivered via retroductal cannulation to the SMGs of 6–8-week-old female C57BL/6J mice (obtained from The Jackson Laboratory; Bar Harbor, ME, USA). BMP6-overexpressing C57BL/6J mice were housed in a pathogen-free animal facility and in compliance with the NIH Guidelines on Use of Animals in Research. All procedures involving animals were performed in an internationally accredited vivarium following institutional guidelines and standard operating procedures. Treatment with ALK2/3 inhibitors, as described herein, was approved by the University of Florida’s Institutional Animal Care and Use Committee and Institutional Biosafety Committee.

After the final saliva collection, BMP6-overexpressing mice and C57BL/6.NOD-*Aec1Aec2* mice were euthanized 2 days later and serum, SMGs, and lacrimal glands (C57BL/6.NOD-*Aec1Aec2* mice only) were collected and stored for histological and immunological analyses as described previously^3,4,22^.

### Adeno-associated viral delivery of BMP6 to the salivary gland

Recombinant adeno-associated viral (rAAV5) vector encoding GFP or BMP6 were prepared as previously described^3^; these vectors are hereafter termed “AAV5-GFP” and “AAV5-BMP6”, respectively. Retrograde ductal instillation was used to deliver 10^11^ particles/mouse (5 × 10^10^ particles/gland) of AAV5-GFP or AAV5-BMP6 in 100 µL PBS with 10 mM MgCl were delivered to the SMGs of 6–8-week-old C57BL/6J mice^3^.

### *In vivo* treatment with ALK2/3 inhibitors

Inhibitors of ALK2/3 (LDN-193189 and LDN-212854) for *in vivo* studies were obtained from Sigma and Dr. Paul Yu (Harvard Medical School)^13^. For studies with BMP6-overexpressing C57BL/6J mice, LDN-193189 was reconstituted in 0.5 mg/mL citrate saline (pH 3.1), and 0.1 mL/mouse (2.5 mg/kg) was injected intraperitoneally (i.p.) twice daily for 3 days. For C57BL/6.NOD-*Aec1Aec2* mice, LDN-212854 or LDN-193189 was reconstituted in 0.55 mg/mL solution of 1.15 mM NaOH PBS; 2.5 mg/kg LDN-212854 or LDN-193189 was injected i.p. daily for 24 days.

### Regulatory volume decrease measurement

Cell membrane water permeability in the HSG cells was measured using a regulatory volume decrease (RVD) assay, which is a measure of rapid transient osmotic swelling via the movement of water through aquaporin channels in response to addition of hypo-osmotic solutions and hypotonic stress^4^. Briefly, 4 × 10^5^ HSG cells per well were cultured with/without 6 ng/mL BMP6. For the indicated cultures, 0.1, 1.0, or 10 nM of LDN-212854 or LDN-193189 was added to the medium, after which the cells were cultured for 4 days. RVD was induced by the addition of 150 mOsm hypotonic solution (HTS), and the change in cell volume was measured before and after HTS stimulation as described previously^3,4,23^.

### *In vivo* salivary flow measurements in mice

Pilocarpine-stimulated salivary flow rate (SFR) was determined in C57BL/6.NOD-*Aec1Aec2* mice and BMP6-overexpressing mice as described previously^3,22^.

### Detection of autoantibodies

Serum was tested for the presence of autoantibodies against SSA/Ro or SSB/La and for antinuclear antibodies (ANA) as described previously^24^.

### Assessment of salivary gland lymphocytic infiltration

Minor SG focus score (FS) of pSS patients was assessed by the SICCA consortium as described previously^25^. Whole mouse SMGs were surgically removed following euthanasia (see above). Tissue sections were stained with hematoxylin and eosin (H&E) stain and lymphocytic infiltration areas in human and mouse SMG H&E slides were captured and assessed using Aperio digital technology (Leica Biosystems, Buffalo Grove, IL, USA), as described previously^24^. Unstained sections were used for immunofluorescent labeling as described previously^3^.

### Immunofluorescent labeling and protein expression quantification

Sections prepared from formalin-fixed paraffin-embedded minor SG (N = 4) and parotid SG tissues (N = 1) from age- and gender-matched healthy volunteers (HVs) were obtained from the NIDCR Sjögren’s Syndrome Clinic or through the cooperative human tissue network. Paraffin-embedded human minor SG slides from N = 79 pSS patients were obtained from the SICCA International Sjögren’s Syndrome Biorepository and Data Registry^25^. The level of BMP6 expression in minor SG tissue sections from pSS patients and healthy volunteer (HV) was determined. (Sample size calculations (https://clincalc.com/stats/samplesize.aspx) based on preliminary differences in signal intensity between pSS patient and HV samples with a Student’s *t* test for unequal variances showed that 14 samples would be sufficient to have at least 80% power to find differences with a 5% level of significance). Positive BMP6 expression in SGs was defined as the relative immunofluorescent intensity in the tested slide ≥ average relative immunofluorescent intensity from the HV slide +2SD (142.3 fluorescence units).

Formalin-fixed, slides were deparaffinized, followed by ethylenediaminetetraacetic acid (EDTA) microwave antigen retrieval^3,4^. For cystic fibrosis transmembrane conductance regulator (CFTR) labeling, Antigen Retrieval Citrate Buffer (pH 9.0) (Abcam, Cambridge, MA, USA) was used. Slides were then blocked with 5% donkey serum (Jackson ImmunoResearch, West Grove, PA, USA) in 0.5% BSA (Sigma-Aldrich Corp.) in PBS at room temperature in a humidified chamber for 30 min then incubated at 4 °C overnight with 100 µL of 10 µg/mL primary antibody solution as listed. For human minor SG and parotid SG samples: mouse anti-human BMP6 (Abcam) goat anti-human activin/ALK-2, goat anti-human BMPR-IA/ALK-3, rabbit anti-human BMPR-II antibodies (R&D Systems) were used; mouse, goat, goat and rabbit ChromPure IgG (Jackson ImmunoResearch) were used as control for BMP6, activin/ALK-2, BMPR-IA/ALK-3 and BMPR-II staining, respectively. For mouse SMG samples: rabbit anti-mouse pSMAD1/5/8 (Invitrogen, Grand Island, NY, USA), rabbit anti-mouse ID3 (Abcam), rabbit anti-mouse AQP5 (Alomone Labs, Jerusalem, Israel), rabbit anti-mouse transmembrane member 16 A (TMEM16A)/anoctamin-1 (ANO1), rabbit anti-mouse Na-K-Cl cotransporter 1 (NKCC1) (a generous gift from Dr. Jim Turner, NIDCR, NIH), rabbit anti-CFTR (Mybiosource, San Diego, CA, USA) antibodies were used; rabbit ChromPure IgG (Jackson ImmunoResearch) was used as control. Slides were then washed in five changes of PBS for 5 min each, and incubated with a 1:200 dilution of 2 mg/mL Alexa Fluor 488- or 596-conjugated goat anti-rabbit or mouse IgG (Abcam) secondary antibody at room temperature in the dark for 1 h, followed by washing in five changes of PBS for 5 min each and counterstaining with DAPI-containing Fluoro-Gel II Mounting Medium (Electron Microscopy Sciences, Hatifield, PA).

To prepare HSG cell slides, cells were immobilized on sterile Nunc Lab-Tek Chamber Slides (Sigma-Aldrich Corp.), and cultured in 1X Minimum Essential Medium (Gibco, Gaithersburg, MD) with 10% FBS containing 1% antibiotics-antimycotic (penicillin, streptomycin, Amphotericin B) (Invitrogen, Frederick, MD) at 37 °C with 5% CO_2_ for 48 h. Next, slides were removed from media and washed twice with PBS, followed by fixation in ice cold methanol at −20 °C for 6 min. Chamber slides were washed in PBS five times and before immunofluorescent labeling, with the same procedure of blocking, primary and secondary antibody incubation, and DAPI mounting medium as described above for paraffin-embedded human parotid and minor SG slides.

All images were acquired with the Olympus FluoView 1000 (Center Valley, PA, USA). Analysis and quantification of expression were performed using Volocity (Quorum Technologies, Puslinch, Ontario, Canada). Pixel intensities were analyzed between a range of 1500 and 4095. For human minor SG BMP6 detection, two consecutive sections were labeled with mouse anti-human BMP6 antibody or mouse IgG as control, respectively (see above). For each slide labeled with anti-BMP6 antibody, three to five confocal images were acquired using a 40× objective. The level of BMP6 expression in minor SGs from patients or HVs was determined by averaging pixel intensity from 15–20 representative minor SG epithelial areas using the 40× objective for each slide, with subtraction of corresponding control mean IgG intensity for each tissue section. The difference was used to determine BMP6 expression in minor SGs from patients or HVs. For data acquisition and analysis of all BMPRs, TMEM16A/ANO1 and NKCC1, three to five confocal images were acquired using a 40× objective. Average pixel intensity for each slide was used to determine expression of the corresponding protein. For AQP5 and CFTR in mouse SMGs, Z-stacked images were acquired as follows. Voxel volume (µm^3^) for each stacked image was used to determine AQP5 expression in the SMG of each animal. For pSMAD1/5/8 and inhibitor of DNA binding protein 3 (ID3), 8 × 8 and 3 × 3 tiled images were acquired for each slide, respectively, and average pixel intensity of each tiled image was used to determine pSMAD1/5/8 or ID3 expression.

### Flow cytometric analysis of salivary gland-infiltrating cells

SMGs were isolated when mice were euthanized. Single-cell suspensions of leukocytes infiltrating SMGs were prepared as described previously^22^ and were resuspended in RPMI 1640 medium containing 10% FBS, 2 mM L-glutamine, 0.05 mM β-mercaptoethanol to a density of 2 × 10^6^ cells/mL. One million cells were pipetted into individual wells of a 24-well microtiter plate pre-coated with anti-CD3 (10 μg/mL) and anti-CD28 (2 μg/mL (BD Biosciences, Billerecia, MA, USA). Cells were incubated for 5 h with Leukocyte Activation Cocktail containing GolgiPlug (2 μL/mL; BD Biosciences Billerecia, MA, USA). Next, cells were collected, fixed, and permeabilized using the Cytofix kit (BD Biosciences Billerecia, MA, USA).

Flow cytometric analysis was performed with LIVE/DEAD Fixable Aqua Dead Cell Stain Kit (Thermo Fisher Scientific, Waltham, MA) and anti-B220 eFluor450, anti-CD3 PE-Cy7, anti-CD4-APC Alexa Fluor 750, anti-Foxp3 PE-Cy5.5, anti-CD25 APC, anti-IL-4 PE, anti-IL-10 PerCP Cy5.5, anti-IL-17 Alexa Fluor 700, and anti-IFN-γ Alexa Fluor 488 antibody (BD Biosciences, Billerecia, MA, USA). Cells were acquired using LSRFortessa (BD Biosciences, Billerecia, MA, USA) and analyzed by FlowJo software (Tree Star, Inc., Ashland, OR, USA).

### Serum cytokine analysis

Serum samples from C57BL/6.NOD-*Aec1Aec2* mice were analyzed for the presence of TNF-α, IL-1α, IL-1β, IL-2, IL-6, IL-4, IL-5, IL-10, IL-12p70, IL-17A and IFN-γ using an 11-plex Mouse Premixed Multi-Analyte Kit for Magnetic Luminex Assay (R&D Systems, Cat# LXSAMSM). Serum samples from these mice were also analyzed for TGF-β1 levels using two kits; TGF-beta 1 Magnetic Luminex Performance Assay and Magnetic Luminex Performance Assay Base Kit, TGF-β (R&D Systems, Cat# LTGM100 and Cat# LTGM00, respectively).

All assays were performed according to the instructions provided by the manufacturer. Briefly, mean fluorescent intensities were collected on a Luminex-200 instrument (Bio-Rad Laboratories, Hercules, CA, USA) using Bio-Plex Manager software version 6.2 (Bio-Rad Laboratories, Hercules, CA, USA). Standard curves for each cytokine were generated using the premixed lyophilized standards provided in the kits, and cytokine concentrations were determined from the standard curve using a 5-point regression to transform the median fluorescent intensity values into concentrations. Each sample was run in duplicate, and the average of the duplicates was used as the measured concentration. Any value that was below the detection level was listed as “LOD” (limit of detection) as reported by the Luminex kits. Analyses were performed using Data Pro Manager version 1.02 (Bio-Rad Laboratories, Hercules, CA, USA).

### Statistical analysis

Unpaired Student’s *t* tests were used to analyze differences in SFR, lymphocytic infiltration, immune cells and protein expression in SMGs between two groups of mice or between minor SGs from pSS patients and HVs. One-way ANOVA followed by Tukey’s multiple comparison was used to analyze the RVD of HSG cells treated with different concentrations of LDN-212854 or LDN-193189. Spearman’s rank correlation coefficient and Pearson correlation coefficient were used to analyze the correlation between BMP6 expression in minor SGs of pSS patients with their unstimulated whole saliva, FS, or lymphocytic infiltration area, respectively. Linear regression was used to analyze the association between FS and lymphocytic infiltration area in minor SGs obtained from pSS patients.

All analyses were performed with GraphPad Prism statistical software version 4.02 (GraphPad Software Inc., La Jolla, CA, USA); *P* ≤ 0.05 was considered to be statistically significant.

## Results

### Increased BMP6 expression in minor salivary glands of pSS patients is associated with xerostomia and sialadenitis

**Figure 1:**
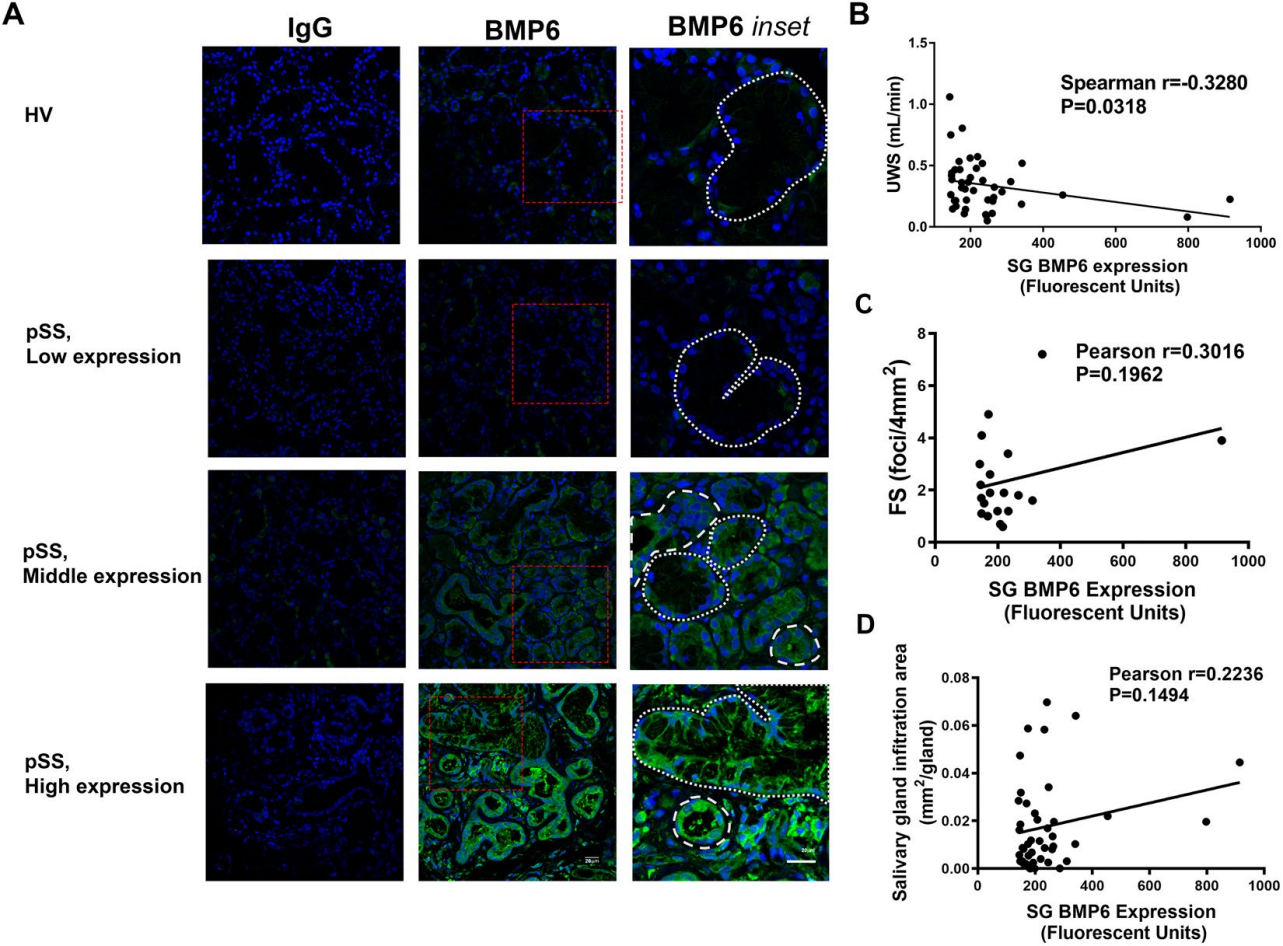
BMP6 expression in minor salivary glands of primary Sjögren’s syndrome patients and correlation with xerostomia and sialadenitis. (**A**) Confocal images demonstrating expression of bone morphogenetic protein 6 (BMP6) in minor salivary glands (SGs) of three representative patients with primary Sjögren’s syndrome (pSS), with either low, middle or high expression (134.0, 261.3, and 797.5 fluorescence units, rows 2–4) and of one healthy volunteer (HV) (112.3 fluorescence units, row 1). Left column: slides labeled with isotype control antibody (mouse IgG) were used as background control (40× objective). Middle column: slides labeled with anti-BMP6 antibody (40× objective). Right row: Inset of the marked areas in the middle row (red dashed box). White large and small dashes were used to mark the ducts and acini tissues, respectively. (**B**) In minor SG, BMP6 positive pSS patients (BMP6 expression ≥142.3 fluorescence units, N = 43), BMP6 expression was negatively correlated with their unstimulated whole saliva (UWS) flow rate (Spearman’s r = −0.328, *P* = 0.0318). (**C**) In minor SG of BMP6 positive pSS patients, BMP6 expression has a trend of positive correlation with focus score (FS) but was not statistically significant (N = 20, only minor SG BMP6 positive patients whose FS data was reported by SICCA were selected. Pearson’s r = 0.3016, *P* = 0.1962). (**D**) In minor SG of BMP6 positive pSS patients, BMP6 expression has a trend of positive correlation with lymphocytic infiltration area (N = 43, Pearson’s r = 0.2236, *P* = 0.1494).

To investigate the relationship between BMP6 expression and clinical manifestations of SS, the level of BMP6 was quantified in sections from minor SG biopsies from a large cohort of female pSS patients (N = 79) (Supplemental Table 1). All patients met the ACR/EULAR 2016 criteria for the diagnosis of pSS^19^. A total of 43 (54.4%) patients showed BMP6 expression levels greater than 2X the standard deviation seen in the epithelia of their minor SGs relative to the HVs (fluorescent intensity +/− SEM 242.8 +/−23.2 vs 90 +/−26.6 *P* < 0.001) (Fig. 1A).

The relationship between minor SG BMP6 expression and two important SG-related manifestations of pSS, SG dysfunction assessed by unstimulated whole saliva (UWS) flow rate and sialadenitis assessed by focus score (FS) or lymphocyte infiltration area was determined. There was a significant negative correlation between minor SG BMP6 expression and UWS flow rate (N = 43, r = −0.33, *P* < 0.05) (Fig. 1B). A trend of positive correlation was found between the minor SG BMP6 expression level and the FS but was not statistically significant (N = 20, r = 0.30, *P* = 0.20) (Fig. 1C). The lymphocytic infiltration area, another reliable measure of focal infiltration in the SG^3,4,24^, was also positively correlated with the BMP6 expression but not statistically significant (N = 43, r = 0.22, *P* = 0.15) (Fig. 1D). In addition, the lymphocytic infiltration area was also significantly associated with the FS in a linear regression model (N = 40, r^2^ = 0.32 *P* = 0.0001) (Supplemental Fig. 1). These findings suggest that minor SG BMP6 expression is associated with the loss of SG function (xerostomia) and with increased inflammation (sialadenitis) in pSS patients.

### ALK2/3 and BMPR2 are expressed in normal human salivary glands

To identify the cell types that may be responsive to BMP6 signaling within the SGs, immunofluorescent labeling and confocal imaging were used to detect the expression of BMP type I receptors ALK2 and ALK3 and the type II receptor BMPR2 in histological sections from the parotid gland of a HV. All three receptors were observed on both ductal and acinar cells (Supplemental Fig. 2), suggesting that both types of cells may be responsive to BMP6 signaling.

**Figure 2:**
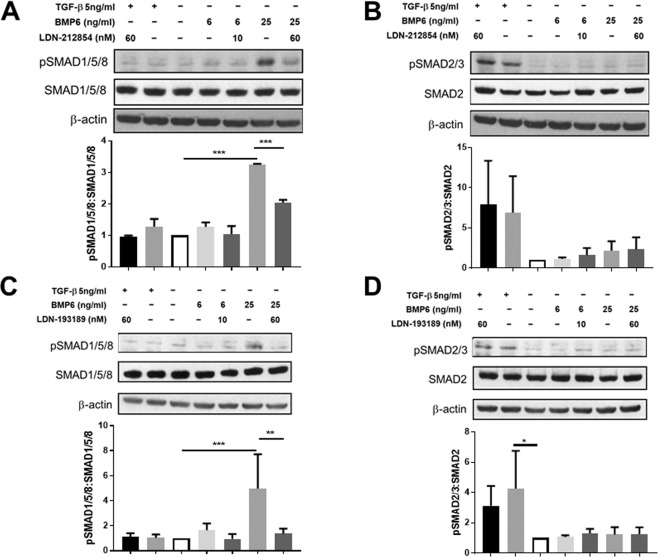
Effect of ALK2/3 inhibitors on increased phosphorylated SMAD1/5/8 and SMAD2/3 expression induced by BMP6 and TGF-β in HSG cells. Phosphorylated SMAD1/5/8 (pSMAD1/5/8), pSMAD2/3, SMAD1/5/8, SMAD2 and β-actin (internal control) expression in HSG cells subjected to bone morphogenetic protein 6 (BMP6) or transforming growth factor-beta (TGF-β) with/without LDN treatment, as measured by Western blot (WB). To determine expression level, fold change of protein expression relative to control cell lysate (HSG cells treated with culture media and diluent for each BMP signaling inhibitor and diluent for LDN only) was used. (**A**) Effect of LDN-212854 on pSMAD1/5/8:SMAD1/5/8 ratio. Upper panel: representative WB of pSMAD1/5/8:SMAD1/5/8 ratio after HSG cells were cultured with 5 ng/mL TGF-β + 60 nM LDN-212854 (lane 1), 5 ng/mL TGF-β (lane 2), control medium (lane 3), 6 ng/mL BMP6 (lane 4), 6 ng/mL BMP6 + 10 nM LDN-212854 (lane 5), 25 ng/mL BMP6 (lane 6), or 25 ng/mL BMP6 + 60 nM LDN-212854 (lane 7). Lower panel: 25 ng/mL BMP6 significantly increased pSMAD1/5/8:SMAD1/5/8 ratio (*P* < 0.0001), but 60 nM LDN-212854 significantly reversed this effect (*P* < 0.0001); 5 ng/mL TGF-β did not change pSMAD1/5/8:SMAD1/5/8 ratio. (**B**) Effect of LDN-212854 on pSMAD2/3:SMAD2 ratio. Upper panel: representative WB of pSMAD2/3:SMAD2 ratio after HSG cells were cultured with different reagents as labeled (see legend A). Lower panel: 5 ng/mL TGF-β increased pSMAD2/3:SMAD2 ratio and 60 nM LDN-212854 did not change this effect; BMP6 with/without LDN-212854 did not alter pSMAD2/3:SMAD2 ratio. (**C**) Effect of LDN-193189 on pSMAD1/5/8:SMAD1/5/8 ratio. Upper panel: representative WB of pSMAD1/5/8:SMAD1/5/8 ratio after HSG cells were cultured with different reagents as labeled (see legend A). Lower panel: 25 ng/mL BMP6 significantly increased pSMAD1/5/8:SMAD1/5/8 ratio (*P* < 0.0001), but 60 nM LDN-193189 significantly reversed this effect (*P* = 0.0004); 5 ng/mL TGF-β did not change pSMAD1/5/8:SMAD1/5/8 ratio. (**D**) Effect of LDN-193189 on pSMAD2/3:SMAD2 ratio. Upper panel: representative WB of pSMAD2/3:SMAD2 ratio after HSG cells were cultured with different reagents as labeled (see legend A). Lower panel: 5 ng/mL TGF-β significantly increased pSMAD2/3:SMAD2 ratio (*P* = 0.0113), but 60 nM LDN-193189 did not change this effect; BMP6 with/without LDN-212193 did not alter pSMAD2/3:SMAD2 ratio. Data shown are means ± SEM from N = 5 (A&C) or N = 2 experiments (B&D). One-way ANOVA followed by Tukey’s multiple comparison was used to compare the seven groups; *P ≤ 0.05, **P ≤ 0.01 and ***P ≤ 0.0001 when compared with control group.

### ALK2/3 inhibitor treatment decreases phosphorylation of SMAD1/5/8 in BMP-stimulated HSG cells

To examine the efficacy of ALK2/3 inhibitors on blocking phosphorylation of SMAD1/5/8 in HSG cells, HSG cells were first treated with BMP6 or TGF-β1 (negative control) and SMAD phosphorylation was examined by Western blotting. As expected, phosphorylation of SMAD1/5/8 was increased by BMP6 stimulation in a dose-dependent manner. Cells treated with 25 ng/mL of BMP6 showed a significant increase in pSMAD1/5/8 (fold change, mean ± SEM, 3.3 ± 0.0 and 5.8 ± 1.2 in experiment of LDN-212854 and LDN-193189, respectively) compared with untreated cells (Fig. 2A,C). However, addition of 60 nM of LDN-212854 or LDN-193189 resulted in a significant decrease in BMP6-induced SMAD1/5/8 phosphorylation compared with BMP6-treated control cells (fold change, mean ± SEM, 2.0 ± 0.1 and 1.4 ± 0.2 for LDN-212854 and LDN-193189, *P* < 0.0001 and *P* < 0.01, respectively;) (Fig. 2A,C). While treatment of cells with TGF-β1 increased phosphorylation of SMAD2/3, pre-treatment of the cells with either of the selective small-molecule BMP signaling inhibitors had no effect on the level of pSMAD2/3 (Fig. 2B,D). Taken together, these results suggest that LDN-212854 and LDN-193189 selectively inhibit SMAD1/5/8 phosphorylation in HSG cells, which is in agreement with previous *in vitro* kinase inhibitor experiments^13^.

### ALK2/3 inhibition blocks BMP6-induced loss of cell membrane water permeability *in vitro*

**Figure 3:**
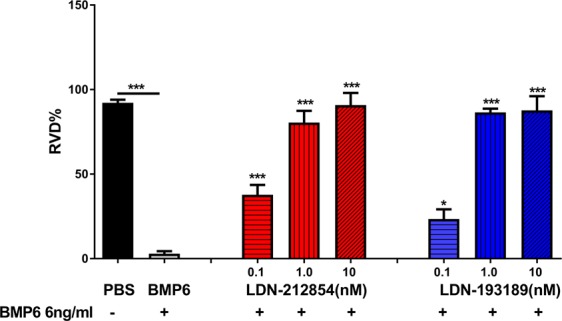
Effect of ALK2/3 inhibitors on regulatory volume decrease in HSG cells. HSG cells were placed in hypotonic solution in absence (black column) or presence of 6 ng/mL bone morphogenetic protein 6 (BMP6) (gray column), 6 ng/mL BMP6 + 0.1, 1.0 or 10 nM LDN-212854 (red columns), or 6 ng/mL BMP6 + 0.1, 1.0 or 10 nM LDN-193189 (blue columns). BMP6 significantly inhibited recovery of cell volume change (as indicated by reduced regulatory volume decrease [RVD%]), but LDN treatment reversed this effect in a dose-dependent manner. Data shown are means ± SEM from N = 3 experiments. One-way ANOVA followed by Tukey’s multiple comparison was used for comparison; *P ≤ 0.05, **P ≤ 0.01 and ***P ≤ 0.0001 when compared with control BMP6-treated group (column 2).

Previous studies showed that treatment of HSG cells with recombinant BMP6 inhibits AQP5 expression, resulting in decreased cell membrane water permeability, as measured by a reduction in regulatory volume decrease (RVD)^4^. To investigate whether inhibition of BMP6 signaling can block the BMP6-induced loss of RVD, HSG cells were treated with 6 ng/mL of BMP6, with or without LDN-212854 or LDN-193189. BMP6 treatment of HSG cells significantly reduced the RVD (mean ± SEM) compared with PBS-treated control HSG cells (2.93 ± 0.61 vs 92.17 ± 1.83%, respectively; *P* < 0.01) (Fig. 3). However, addition of LDN-212854 (0.1, 1.0 or 10 nM) inhibited the RVD loss induced by BMP6 in a dose-dependent manner (37.80 ± 5.83, 80.50 ± 6.93 and 90.83 ± 7.17%, respectively vs 2.93 ± 0.61 in BMP6 only-treated cells; all *P* < 0.05). A dose-dependent increase in the RVD was also measured upon treatment with the same three doses of LDN-193189 (23.40 ± 8.80, 86.50 ± 2.21 and 87.70 ± 8.42%, respectively; all *P* < 0.05 compared with BMP6 only-treated cells). Moreover, the RVD of HSG cells treated with either LDN-212854 or LDN-193189 at both 1.0 and 10 nM was not significantly different from that of PBS-treated HSG cells (all *P* > 0.05). These findings suggest that treatment with LDN-212854 or LDN-193189 can block BMP6-induced loss of the RVD *in vitro* in the HSG cells.

### Inhibition of ALK2/3 restores salivary gland function in BMP6-overexpressing mice and C57BL/6.NOD-*Aec1Aec2* mice

Based on our finding that BMP signaling inhibitors blocked BMP6-induced RVD loss, we hypothesized that *in vivo* treatment with these small molecules might restore SG function in animal models of pSS. We tested this possibility in two SS animal models: C57BL/6J mice overexpressing BMP6 specifically in the SMGs^3^, and C57BL/6.NOD-*Aec1Aec2* mice^20,21^.

**Figure 4:**
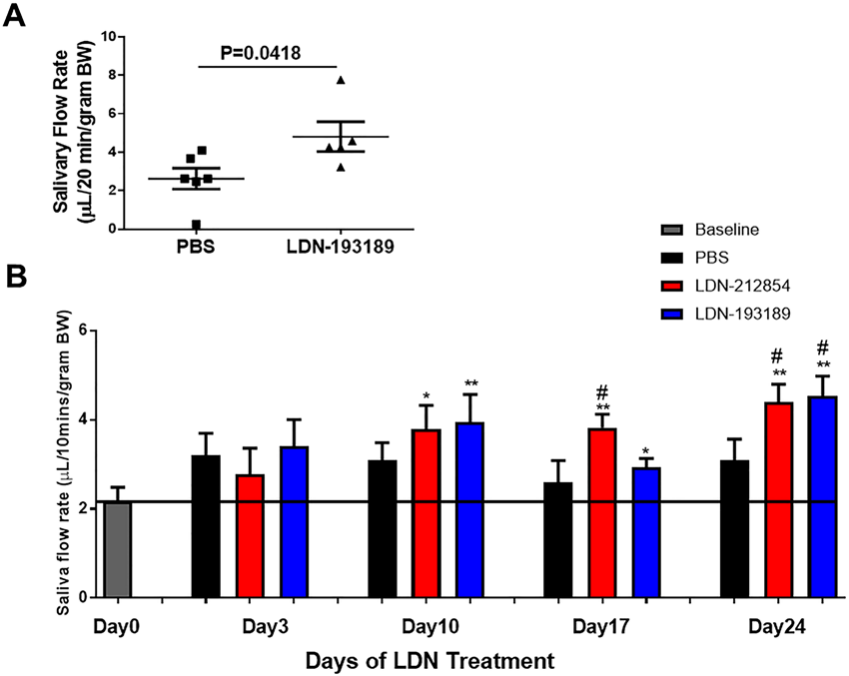
Saliva secretion in BMP6-overexpressing C57BL/6J mice and C57BL/6.NOD-*Aec1Aec2* mice after ALK2/3 inhibitor treatment. (**A**) Submandibular glands (SMGs) of female C57BL/6J mice were cannulated and AAV5 vector encoding bone morphogenetic protein 6 (BMP6) was instilled to promote local BMP6 expression. Twenty-four months post-cannulation, mice were treated with citrate saline or 2.5 mg/kg LDN-193189 administered i.p. twice daily for 3 days. LDN-193189–treated mice showed a significant increase of salivary flow rate (SFR) compared with saline-treated mice. Data shown are means ± SEM and were analyzed with unpaired Student’s *t* test. (**B**) Male (M) and female (F) C57BL/6.NOD-*Aec1Aec2* mice with established disease were treated daily with PBS (black columns, N = 13, 7M6F), 2.5 mg/kg LDN-212854 (red columns, N = 13, 6M7F), or 2.5 mg/kg LDN-193189 (blue columns, N = 14, 8M6F) for 24 days. SFR was determined in all mice prior to LDN treatment (day 0, baseline), and on day 3, 10, 17 and 24 thereafter. SFR significantly increased in LDN-treated mice compared with PBS-treated mice (control group) from day 10 to 24. Data shown are means ± SEM. Unpaired Student’s *t* test was used to compare two groups. *P < 0.05 and **P < 0.01 compared with baseline, ^#^*P* < 0.05 compared with PBS-treated group at same time point.

Salivary hypofunction was induced in C57BL/6J mice by retroductal instillation of recombinant adeno-associated virus type 5 (AAV5) vectors encoding BMP6, as reported previously^3^. These animals develop persistent reduced salivary flow with mild but significantly increased lymphocytic infiltration^3^. Four weeks after vector delivery, the pilocarpine-stimulated salivary flow rate (SFR; mean ± SEM) was decreased approximately two-fold compared with that of AAV5-GFP–treated control mice (AAV5-BMP6: SFR: 1.3 ± 0.3 vs AAV5-GFP: SFR: 3.1 ± 0.7 µL/g body weight in 20 min; *P* < 0.05) (Supplemental Fig.  3A). A statistically significant decrease in SG function was still detectable twenty-four months after retroductal cannulation with AAV5-BMP6 compared with the age-matched AAV5-GFP control group (AAV5-BMP6: SFR: 1.9 ± 0.3 vs AAV5-GFP: SFR: 3.6 ± 0.4 µL/g body weight in 20 min; *P* < 0.001) (Supplemental Fig.  3B). To test the effect of ALK2/3 inhibition on restoring SG function, AAV5-BMP6–treated mice with decreased SFR were randomly divided into two groups and treated with 0.1 mL of citrate saline daily (control) or 0.1 mL of 2.5 mg/kg LDN-193189 twice daily, for 3 days. Treatment with LDN-193189 significantly increased the SFR in AAV5-BMP6–treated mice compared with that in the saline control group (AAV5-BMP6 + LDN-193189: SFR: 4.81 ± 0.77 vs AAV5-BMP6 + saline: SFR: 2.62 ± 0.54 µL/g body weight in 20 min; *P* < 0.05) (Fig. 4A).

Similar results were observed in C57BL/6.NOD-*Aec1Aec2* mice, which are a model of spontaneous development of SS. Mice with established disease (34–38 weeks of age) were randomly divided into three groups and treated daily with either PBS, 2.5 mg/kg LDN-212854, or 2.5 mg/kg LDN-193189 for 24 days. There was a significant increase in SFR in both LDN-treated groups after 10 days of treatment compared with the baseline SFR (3.80 ± 0.53 and 3.90 ± 0.62 µL/g body weight in 10 min for LDN-212854– and LDN-193189–treated groups, 2.18 ± 0.31 for baseline respectively; both *P* < 0.05) (Fig. 4B**)**. The recovery of pilocarpine-stimulated salivation persisted through the end of the experiment (day 24) (4.40 ± 0.40 and 4.54 ± 0.45 µL/g body weight in 10 min in LDN-212854– and LDN-193189–treated groups, respectively; both *P* < 0.01 compared with baseline SFR). By day 24, a significant increase in SFR was also observed in both LDN-treated groups compared with the PBS-treated control group (3.10 ± 0.47 µL/g body weight in 10 min; both *P* < 0.05) (Fig. 4B).

The restoration of salivary gland flow in SMG BMP6-overexpressing C57BL/6J mice and C57BL/6.NOD-*Aec1Aec2* mice suggests that treatment with an ALK2/3 inhibitor can restore salivary gland function *in vivo*.

### Inhibition of ALK2/3 decreases phosphorylation of SMAD1/5/8 and induction of inhibitor of DNA binding protein 3 expression in C57BL/6.NOD-*Aec1Aec2* mice

**Figure 5:**
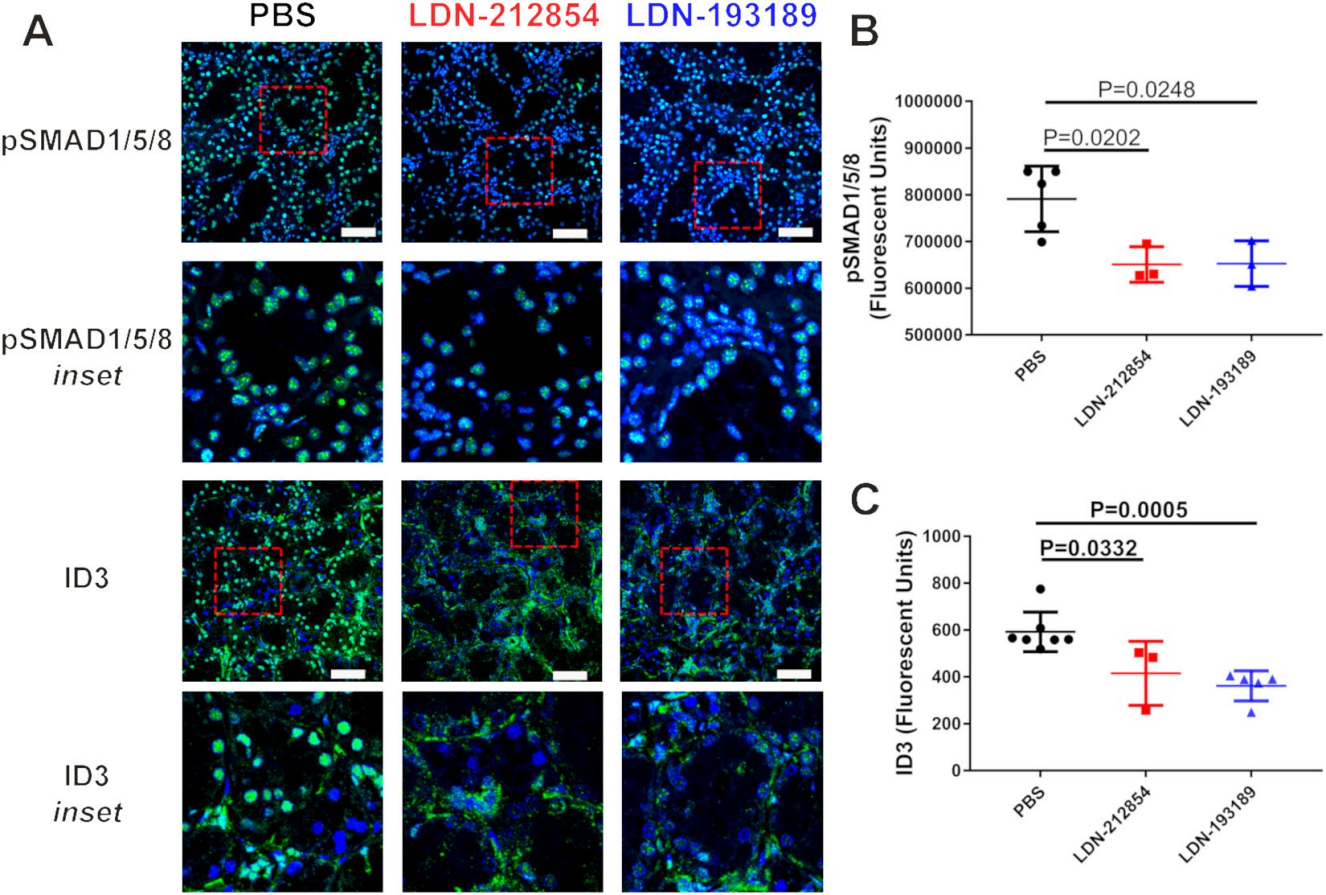
pSMAD1/5/8 and ID3 expression in submandibular glands of C57BL/6.NOD-*Aec1Aec2* mice after ALK2/3 inhibitor treatment. Confocal images demonstrating expression of pSMAD1/5/8 and ID3 in submandibular glands (SMGs) of LDN-treated C57BL/6.NOD-*Aec1Aec2* mice. (**A**) Representative images of pSMAD1/5/8 and ID3 expression in SMGs of mice daily treated with PBS (control, left panels), 2.5 mg/kg LDN-212854 (middle panels), or 2.5 mg/kg LDN-193189 (right panels) for 24 days. Row 1 & 3: slides labeled with pSMAD1/5/8 and ID3 antibodies, respectively (40× objective, white bar: 50 µm). Row 2 & 4: Inset for the marked area in row 1 and 3, respectively (red-dashed box). (**B**,**C**) Statistical analysis showed a significant decrease in (**B**) pSMAD1/5/8 and (**C**) ID3 expression in LDN-treated mice compared with PBS-treated (control) mice (*P* < 0.05). Data shown are means ± SEM from N ≥3 mice respectively. Statistical significance was determined with unpaired Student’s *t* test.

Our *in vitro* experiments indicated that LDN-212854 and LDN-193189 inhibit phosphorylation of SMAD1/5/8 in HSG cells (Fig. 2). To investigate whether LDN inhibitors can block phosphorylation of SMAD1/5/8 in salivary glands and affect pSMAD1/5/8 responsive genes *in vivo*, confocal imaging was used to detect changes in the phosphorylation of SMAD1/5/8 and the expression of inhibitor of DNA binding protein 3 (ID3; a pSMAD1/5/8 responsive gene), in SMG from LDN-treated C57BL/6.NOD-*Aec1Aec2* mice. Although many genes are responsive to BMP6 signaling via pSMAD1/5/8, ID3 is well established BMP downstream effector^26^. Confocal tiled images were used to measure the total immunofluorescent intensity over a 2-mm^2^ area. The expression of pSMAD1/5/8 (mean ± SD) in SMG was significantly decreased in LDN-treated mice compared with PBS-treated control mice (LDN-212854: 6.5 × 10^5^ ± 2.2 × 10^4^; LDN-193189: 6.5 × 10^5^ ± 2.8 × 10^4^; and PBS: 7.9 × 10^5^ ± 3.1 × 10^4^ relative fluorescence units; both *P* < 0.05) (Fig. 5A upper row & B). Similarly, ID3 nuclear expression (mean ± SD) in SMG was significantly decreased in LDN-212854– (4.2 × 10^3^ ± 78.9 relative fluorescence units) and LDN-193189–treated mice (3.6 × 10^3^ ± 28.6 relative fluorescence units) compared with PBS-treated mice (5.9 × 10^3^ ± 32.0 relative fluorescence units; both *P* < 0.05) (Fig. 5A lower row & C). The decrease in SMG pSMAD1/5/8 and ID3 in response to LDN ALK2/3 inhibitor treatment *in vivo* is in agreement with the previous *in vitro* treatment results in HSG cells and supports a mechanism of action for the recovery of salivary gland secretion.

### Inhibition of ALK2/3 decreases the number of Th1 cells in the submandibular glands of C57BL/6.NOD-*Aec1Aec2* mice

C57BL/6.NOD-*Aec1Aec2* mice are similar to pSS patients in that they develop an autoimmune disease with localized inflammation in secretory epithelia especially in salivary and lacrimal glands, and increased production of autoantibodies and proinflammatory cytokines, such as IFN-γ^20,21^. After treatment with ALK2/3 inhibitors, serum, SMGs, and lacrimal glands were collected from C57BL/6.NOD-*Aec1Aec2* mice and tested for changes in the salivary gland and systemic immune system. No significant changes in serum levels of antinuclear antibodies or of the SS-associated autoantibodies directed against SSA/Ro and SSB/La were detected in LDN-treated mice compared with the PBS-treated control group (data not shown). No significant changes in the size of the lymphocytic infiltrate were noted in the SMGs and lacrimal glands of LDN-treated C57BL/6.NOD-*Aec1Aec2* mice compared with PBS-treated controls (Supplemental Fig. 4). However, there was a greater than two-fold decrease in CD4^+^IFN-γ^+^ T cells in the SMGs of LDN-212854- and LDN-193189- treated mice compared with those from PBS-treated control mice, as measured by flow cytometry (N = 6 (3 males and 3 females)/group, *P* < 0.05) (Table 1, Supplemental Fig. 5). No significant changes in the percentage of CD19^+^ B cells or other T cell populations were detected (Table 1). These data suggest that although LDN-212854 or LDN-193189 treatment does not change the size of the area of lymphocytic infiltration, ALK2/3 inhibition decreases the relative number of proinflammatory Th1 cells in SMGs.

**Table 1:** Flow cytometry analysis of the SMG lymphocytic infiltrated cells from the C57BL/6.NOD-Aec1Aec2 mice (N = 6 mice (3 males and 3 females)/group.

	B cells	T cells		
	CD19+	CD3+ CD4+	CD4+ IFN-γ+	CD3+ CD4+
				CD25+ Foxp3+
**Lymphocyte % (Mean** ± **SD)**				
PBS	0.68 ± 0.51	29.79 ± 13.86	42.10 ± 12.57	1.84 ± 1.16
LDN212854	3.22 ± 3.49	28.03 ± 10.93	18.18 ± 4.07*	1.00 ± 0.94
LDN193189	2.52 ± 2.62	28.07 ± 13.57	18.02 + 4.75*	1.98 ± 1.47
**P value (t-test)**				
PBS vs LDN212854	0.1224	0.8525	0.0014*	0.9609
PBS vs LDN193189	0.1082	0.7609	0.0013*	0.9074
LDN212854 vs LDN193189	0.7006	0.8803	0.9492	0.0339

*P < 0.05.

### ALK2/3 inhibition alters serum cytokines in C57BL/6.NOD-*Aec1Aec2* mice

To investigate the effects of ALK2/3 inhibitors on the systemic immune system, a multiplex assay was used to measure serum cytokines. From data analysis, three different cytokine levels were found to be altered. First, a statistically significant decrease (p < 0.05)) in serum IL-1β level was observed between the LDN193189 treated C57BL/6.NOD-*Aec1Aec2* mice (106.1 ± 2.2 pg/mL) compared to the PBS control group (118.7 ± 5.9; (Supplemental Fig. 6A). Both LDN-212854– and LDN-193189–treated mice also showed a significant decrease in the IL-2 level (mean ± SEM) compared with PBS-treated mice (2.60 ± 0.23 and 3.59 ± 0.24 vs 2.41 ± 0.19 pg/mL for LDN-212854, LDN-193189 vs PBS, respectively; *P* < 0.05) (Supplemental Fig. 6B). LDN-193189–treated mice had a significantly increased TGF-β1 level (mean ± SEM) compared with PBS-treated mice (267 ± 40 vs. 123 ± 21 ng/mL, respectively, *P* < 0.01); LDN-212854-treated mice showed an increase in TGF-β1 level (200 ± 43 vs 123 ± 21 ng/mL; *P* = 0.13, but the difference with PBS-treated mice was not significant (Supplemental Fig. 6C). Overall, the changes in serum cytokine expression suggest that inhibition of ALKL2/3 results in a decrease in proinflammatory cytokines and an increase in a cytokine (TGF-β1) associated with regulatory T cells.

**Figure 6:**
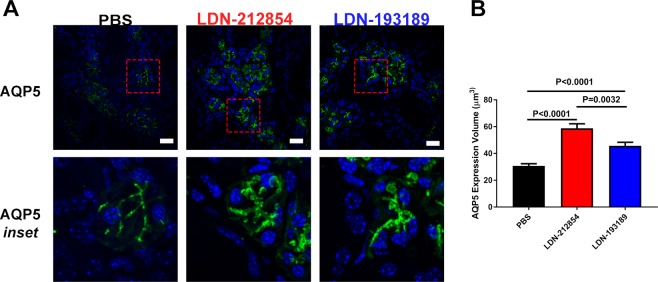
Expression of aquaporin 5 in submandibular glands of C57BL/6.NOD-*Aec1Aec2* mice. Confocal images of submandibular gland (SMG) tissue of LDN-treated C57BL/6.NOD-*Aec1Aec2* mice. (**A**) Representative Z-stack images from SMG slides labeled with isotype control antibody anti-mouse AQP5 antibody for mice treated with PBS (left column), LDN-212854 (middle column) or LDN-193189 (right column). Row 1: slides labeled with AQP5 antibody (40× objective, white bar: 20 µm). Row 2: Inset for the marked area in row 1 (red-dashed box) (N ≥ 3). (**B**) Statistical analysis of AQP5 expression measured by total volume from Z-stack images in panel A showed a significant increase in AQP5 expression in LDN-treated mice compared with control mice (*P* < 0.0001). Data shown are means ± SEM. Statistical significance was determined with unpaired Student’s *t* test.

### LDN-212854 and LDN-193189 alter AQP5 protein expression and localization in C57BL/6.NOD-*Aec1Aec2* mice

Initiation of fluid secretion from the SG requires movement of water and ions across the membranes of acinar cells. AQP5 is the main water channel for salivary fluid secretion^4,27^. Decreased AQP5 expression has been observed in minor SGs of pSS patients by microarray analysis, and an altered distribution of AQP5 has been described in SS patients and in mice overexpressing BMP6 in their SGs by histological studies^3^. In addition, in pSS patients who respond to treatment with rituximab, AQP5 expression increases^28^. Gene delivery of another water channel, AQP1, to C57BL/6.NOD-*Aec1Aec2* mice can rescue the loss of SG function in these mice^4^. These results suggest a central role of AQP-mediated membrane water permeability in SG function.

To determine whether the increase in SG function by ALK2/3 inhibitor treatment is associated with a change in AQP5 expression, SMG sections from LDN-treated and control C57BL/6.NOD-*Aec1Aec2* mice were imaged by immunofluorescent labeling and confocal microscopy. A significant increase in AQP5 expression (mean ± SEM) was observed in the SMGs from both LDN-212854– (58.80 ± 3.40 µm^3^) and LDN-193189–treated mice (45.73 ± 2.63 µm^3^) compared with the PBS-treated group (30.71 ± 1.67 µm^3^; both *P* < 0.0001) (Fig. 6). Confocal imaging analysis of the expression of other proteins that are important for salivary function, including transmembrane member 16A (TMEM16A/ANO1) and Na-K-Cl cotransporter 1 (NKCC1), showed no significant changes in LDN-treated mouse SMGs compared with the control group (Supplemental Fig. 7). LDN-193189 treatment resulted in complete relocalization of the cystic fibrosis transmembrane conductance regulator (CFTR), an apical cell membrane chloride channel, from vesicles to the apical membrane of SMG epithelial cells. Although aggregation of CFTR was still observed following treatment with LDN-212854 (Supplemental Fig. 7) some relocalization was detected, which maybe sufficient for a functional effect. The difference in CFTR response is likely a result of the broader effects of LDN-193189 on both ALK2 and ALK3 compared to LDN-212854. These results support a central role of increased AQP5 levels in salivary gland function in response to ALK2/3 inhibition in models of Sjögren’s syndrome.

## Discussion

Although increased interferon-alpha signaling is well established in SS^29^, little is known about alterations in other cell signaling pathways. We recently discovered by unbiased microarray mRNA analysis that BMP6, an important cytokine, is up-regulated in the salivary glands of a subset of SS patients and confirmed in mice the association between overexpression of BMP6 and the loss of fluid secretion and increased the inflammatory filtrate within the salivary glands^3^. Here we extended our studies by analyzing BMP6 expression in an independent SS cohort and confirming that increased BMP6 salivary gland protein expression occurs in approximately 54% of the SS subjects compared to case controls. Importantly, the increased expression of BMP6 protein expression seen in SS cases significantly correlated with decreased salivary flow rate. Previous expression of BMP6 in mice showed a statistically significant increase in focus score, but only a trend towards a positive correlation with size of lymphocytic infiltration was detected in patient samples. This may reflect the broader range of expression that is possible to achieve in mice experiments or a threshold of activation effect required for an increase in focus score. In addition, immunostaining confirmed BMP6 responsive receptor expression on both acinar and ductal cell types within human salivary glands laying the groundwork for the rationale targeting of the BMP-6 signaling pathway for the treatment of SS.

Using cell culture and mouse models of SS, we studied whether targeted pharmacological blockade of BMP6 signaling might attenuate autoimmune disease activity. For these experiments, selective ATP competitive small molecule kinase inhibitors of the BMP downstream signaling activity were utilized. Two inhibitors, LDN-212854 and LDN-193189, have IC_50_ for BMP responsive ALK receptors in the low nM range when compared to TGF-β/activin responsive ALK receptors. Treatment of HSG cells with either LDN-212854 or LDN-193189 decreased the BMP6 induced phosphorylation of SMAD1/5/8 and inhibited the BMP6-induced loss of cell membrane water permeability. *In vivo* treatment with these BMP6 signaling inhibitors in two mouse models of SS restored salivary gland secretion, decreased proinflammatory cells in the gland, decreased cytokines in the serum, and increased levels of TGF-β1, a cytokine often associated with regulatory T cell population. Treatment with LDN-212854 or LDN-193189 also increased AQP5 protein expression and increased salivary secretions in C57BL/6.NOD-*Aec1Aec2* mice. Although salivary gland secretion is complex, water channels such as AQP5 play an important role in the final steps of this process. The physiologic response to ALK2/3 inhibitor treatment implies restoration of water movement through inhibition of the SMAD1/5/8 signaling pathway is a fundamental aspect to loss of salivary gland function and supports the possibility of using ALK2/3 inhibition therapeutically.

Sjögren’s syndrome is often associated with a strong interferon response and IFN-γ producing Th1 cells play an important role in the initiation and development of SS in patients and animal models^4,22,30–32^. A recent study reported that BMP6 upregulated interferon signaling and downregulated inhibitors of interferon signaling, suggesting a direct interacting between the BMP6 and IFN signaling^30^. The present study supports this and suggests ALK2/3 inhibition can regulate the expression of IFN-γ. Further studies will be necessary to test for BMP6 responsive receptors on IFN-producing immune cells to see if ALK2/3 inhibitors alter their specific cytokine production profile in the context of Sjögren’s syndrome. Salivary gland epithelia are reported to be involved in immune regulation as non-professional antigen presenting cells and directly produce pro-inflammatory cytokines such as IFN-γ, IL-17, IL-23 and chemokines (for review, see^31^). As an important pro-inflammatory T cells, IFN-γ producing Th1 cells play an important role in the initiation and development of the pSS^32^. IFN-γ has been suggested as a key effector point in the inflammation and function in models of pSS^4,22^. In addition,IFN-γ induces the expression of SMAD7, an antagonistic SMAD, which prevents the interaction of SMAD3 with the TGF-β receptor. The results indicate a mechanism of transmodulation between the STAT and SMAD signal-transduction pathways, which has been reported previously^33^. A connection between BMPs and proinflammatory cytokines such as IFN-γ is beginning to emerge^34^. In our study, although the inhibition of ALK2/3 didn’t change the infiltrate size or focus score, the observed reduction of IFN-γ producing Th1cells indicated an important decrease in inflammation in the SG and maybe related to the increase in gland secretion.

A key unanswered question remains what is driving the increased mRNA expression of BMP6 in the salivary glands of patients. BMP6 can be produced by many cell types at low levels but its induced overexpression in SS appears to be mainly due to increased transcriptional activity. Additional research will be required to define which cells in the gland are producing the BMP6 and what is the stimulus. Recent work on BMP6 in the liver suggests that in some contexts BMP/SMAD signaling regulates the expression of a broad repertoire of antiviral genes including IRF1, IRF2, IRF7 and IRF9, which in turn regulate many other antiviral genes^30^. Furthermore, it has been shown that BMP6 could enhance an antiviral response to HCV and potently block HCV replication independently of type 1 interferon signaling, suggesting BMP-6 may be interconnected with host antiviral responses.

Many animal models have been developed for studying Sjögren’s syndrome including derivatives of the NOD line, induced models such as Ro or M3R immunized mice, or engineer strains such as STIM1/2 KO, matriptase KO, IL-12 TG, BAFF TG, IL-14α TGPI3K KO, or ID3 KO systemic and T-cell specific models (for review see^35^). Collectively, these models and interventions that have been tested suggest that many cell types (B, T, mesenchymal stem cells, thyroid, epithelial cells, etc.) can be involved in the development of the disease. Therefore, identifying critical pathways in disease development and then formulating broad pathway specific inhibitors is an important progression in the development of novel interventions for complex autoimmune diseases like Sjögren’s syndrome.

Although there are likely alterations in many different signaling pathways in SS, our study focused on BMP6 signaling as one driver of SS inflammation and dysfunction. As pSS is a complex disease, further studies are necessary to identify other pathogenic mechanisms involved in patients with normal levels of BMP6 expression responsible for altered function and inflammation to develop more personalized therapies for this disease. Although our treatment studies were performed in mouse models of SS, our findings suggest the possibility that there might be plasticity within the salivary glands of pSS patients and that treatment altering the BMP6 and other signaling pathways might allow for recovery of the salivary gland function in patients.

## Supplementary information

Supplementary information.
