# Supplementary material for: Inhibition of bone morphogenetic protein 6 receptors ameliorates Sjögren’s syndrome in mice
Source: Sci Rep. 2020 Feb 19;10:2967. doi: 10.1038/s41598-020-59443-z (PMC7031521; doi:10.1038/s41598-020-59443-z)
Supplement: Supplementary file 1 — Supplementary information. [file 41598_2020_59443_MOESM1_ESM.pdf]

Inhibition of bone morphogenetic protein 6 receptors ameliorates Sjögren's syndrome in mice

Hongen Yin[1], Lovika Kalra[1], Zhennan Lai[1], Maria C. Guimaro[1], Lauren Aber[1], Blake M. Warner[1], Drew Michael[1], Nan Zhang[1], Javier Cabrera-Perez[1], Arif Karim[1], William D. Swaim[1], Sandra Afione[1], Alexandria Voigt[2], Cuong Q. Nguyen[2], Paul B. Yu[3], Donald B. Bloch[4] and John A. Chiorini[1]

[1]AAV Biology Section, Division of Intramural Research, National Institute of Dental and Craniofacial Research, National Institutes of Health, Bethesda, MD, USA;

[2]Department of Pathology and Infectious Diseases, University of Florida, Gainesville, FL, USA;

[3]Cardiovascular Division, Brigham and Women's Hospital, Harvard Medical School, Boston, MA, USA.

[4]Center for Immunology and Inflammatory Diseases and the Division of Rheumatology, Allergy, and Immunology of the Department of Medicine, Massachusetts General Hospital, Harvard Medical School, Boston, MA, USA.

Correspondence to: jchiorini@dir.nidcr.nih.gov

Supplemental Table 1. Characteristics of the patients with Primary Sjögren's Syndrome (pSS)

	**pSS (N=79)**	**Normal Range**
Age ([years], mean, min-max)	49.6 (21-74)	
Sex	Female	
Race (White/Hispanic/African American/Asian/Native American)	33/6/9/30/1	
Unstimulated saliva flow ([mL/15minute], mean±SD, min-max)	5.11±3.89 (0.77-30.74)	>1.5mL/15min
Focus score (mean±SD, min-max)	2.0±1.4 (0.2-7.2)	0-1
Anti-Ro/SSA positive	58	Negative
Anti-La/SSB positive	25	Negative
RF	40	Negative
ANA positive	48	Negative

Except where indicated otherwise, values are the number of subjects (N).

Supplemental Figure 1

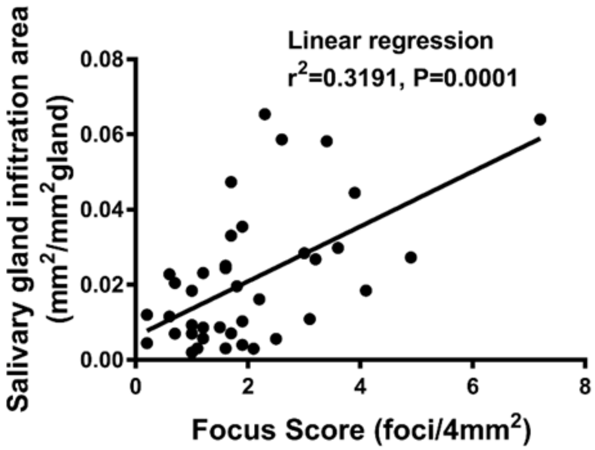

Supplemental Figure 1. Association between focus score and lymphocytic infiltration area in primary Sjögren's syndrome patients

Focus score (FS) was positively associated with lymphocytic infiltration area in minor salivary glands of patients with primary Sjögren's syndrome (N = 40, linear regression: $r^2 = 0.32$, $P < 0.001$).

Supplemental Figure 2

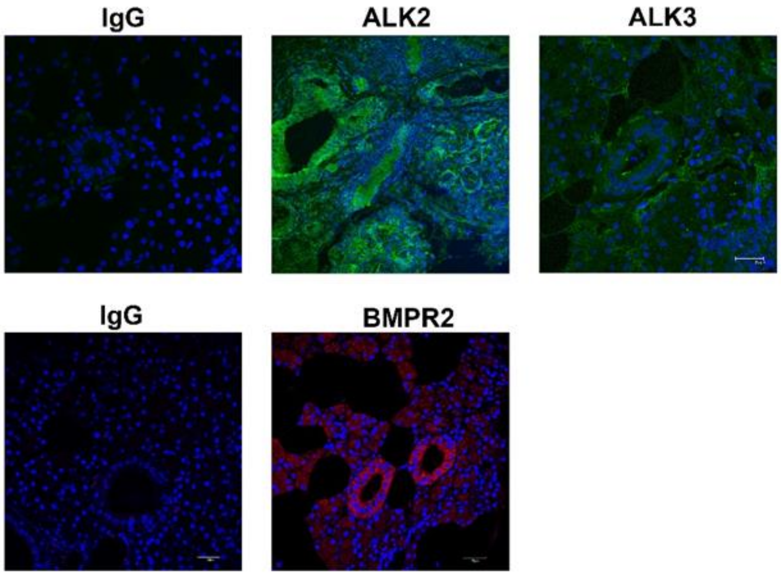

Supplemental Figure 2. Immunofluorescent labeling of BMPR in normal human salivary glands

First row: confocal images demonstrating expression of bone morphogenetic protein (BMP) type

I receptors ALK2 (middle) and ALK3 (right) in parotid gland of a healthy volunteer (HV); control slide was labeled with goat IgG (left). Second row: confocal images demonstrating expression of type II receptor BMPR2 (right) in HV's parotid gland; rabbit IgG was used as control (left).

Supplemental Figure 3

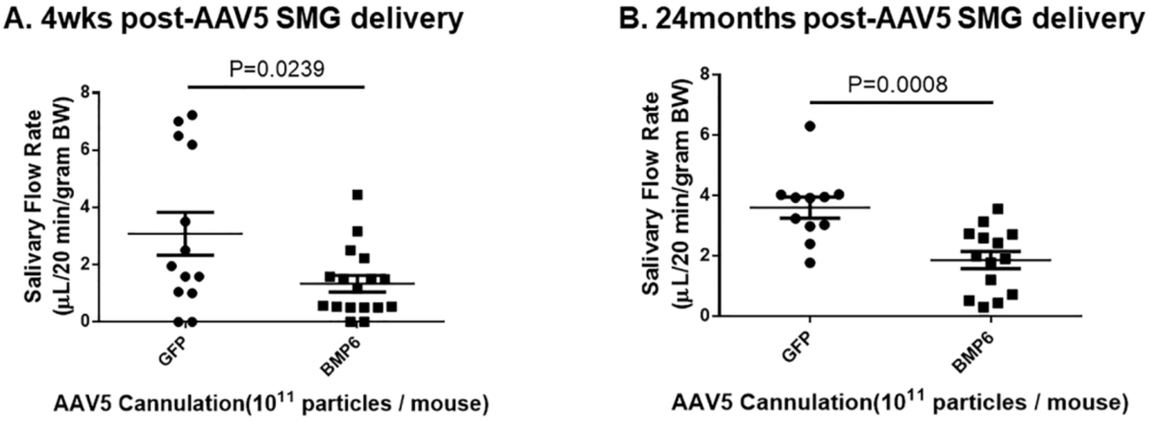

Supplemental Figure 3. Saliva secretion after AAV5-mediated BMP6 overexpression in submandibular glands of C57BL/6J mice

AAV5-GFP or AAV5-BMP6 were delivered to the submandibular glands of female C57BL/6J mice by retroductal cannulation. **A)** Salivary flow rate (SFR) of AAV5-GFP (N = 13, dots) and AAV5-BMP6 mice (N = 17, squares) 4 weeks postcannulation. **B)** SFR of AAV5-GFP (N = 11, dots) and AAV5-BMP6 mice (N = 14, squares) 24 months postcannulation. Data shown are means ± SEM for each group. Statistical significance was determined with unpaired Student's *t* test.

Supplemental Figure 4

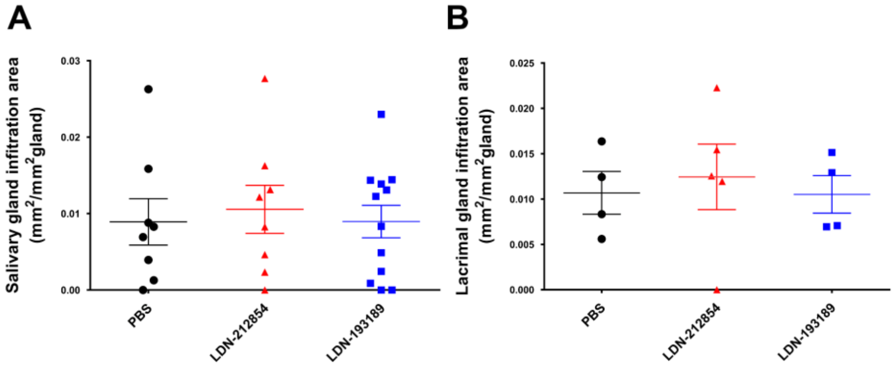

Supplemental Figure 4. Lymphocytic infiltration in exocrine glands of C57BL/6.NOD-

***Aec1Aec2* mice**

Lymphocytic foci were detected by H&E stained sections of submandibular glands (SMGs) and lacrimal glands of C57BL/6.NOD-*Aec1Aec2* mice. Lymphocytic infiltration was quantified as infiltration area (in mm^2) per mm^2 total glandular size on the section. **A)** Lymphocytic infiltration area in SMGs of C57BL/6.NOD-*Aec1Aec2* mice treated with PBS (black dots, N = 8), LDN-212854 (red triangles, N = 8), or LDN-193189 (blue squares, N = 12). **B)** Lymphocytic infiltration area in lacrimal glands of C57BL/6.NOD-*Aec1Aec2* mice treated with PBS (black dots, N = 4), LDN-212854 (red triangles, N = 5), or LDN-193189 (blue squares, N = 4). Data shown are means ± SEM. Statistical significance was determined with unpaired Student's *t* test.

Supplemental Figure 5

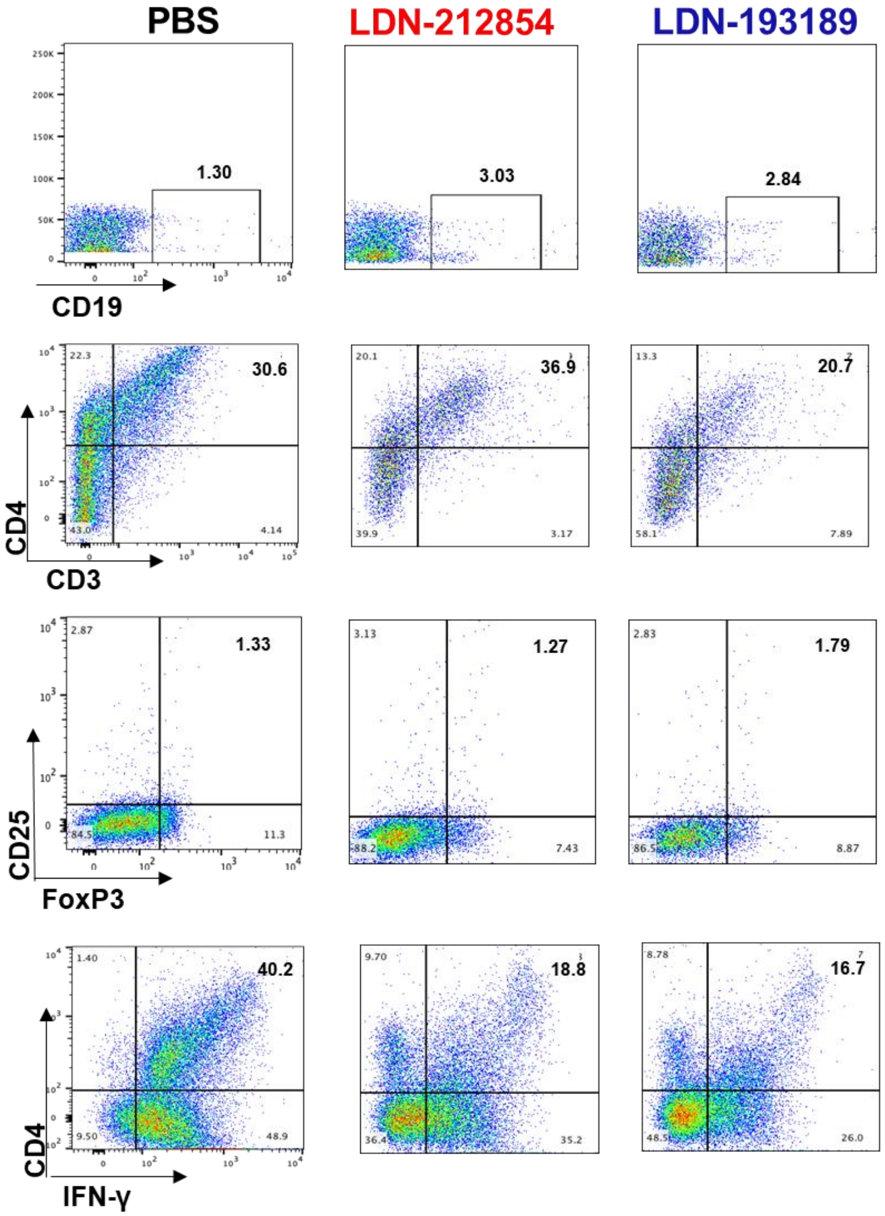

Supplemental Figure 5. Flow cytometry analysis for infiltrated cells in SMG from C57BL/6.NOD-*Aec1Aec2* mice after ALK2/3 inhibitor treatment

Infiltration cells were isolated from SMG of C57BL/6.NOD-*Aec1Aec2* mice treated with PBS (left column), LDN-212-854 (middle column) and LDN-193189 (right column) at the end of the study and analyzed for the indicated B and T cells. First row: CD19+ B cells; Second row: CD3+CD4+ T cells; Third row: CD4+CD25+Foxp3+ Treg cells; Fourth row: CD4+IFN-γ+ Th1 cells.

Supplemental Figure 6

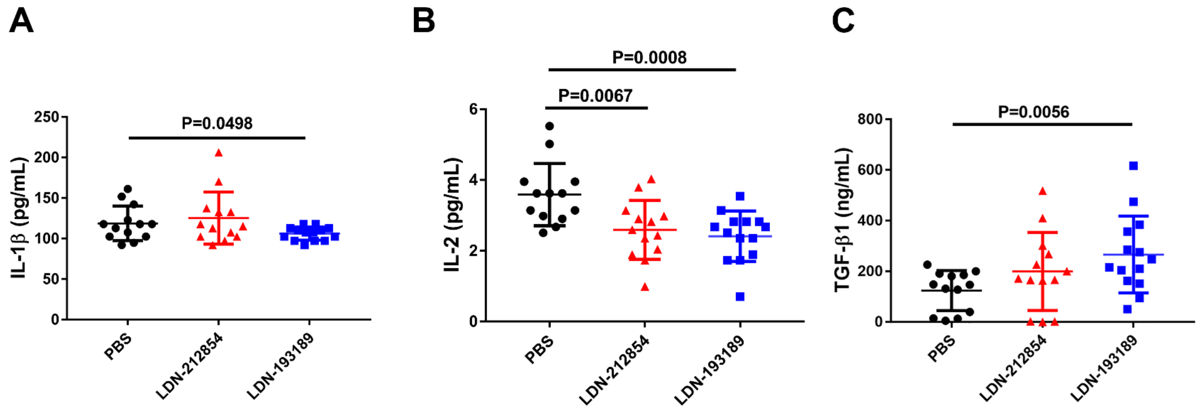

Supplemental Figure 6. Serum cytokines in C57BL/6.NOD-*Aec1Aec2* mice after ALK2/3 inhibitor treatment

Serum samples were collected from C57BL/6.NOD-*Aec1Aec2* mice at the end of the study and analyzed for levels of the indicated cytokines with a multiplex cytokine assay in duplicate. Serum cytokines of **A)** IL-1β, **B)** IL-2 and **C)** TGF-β1 in mice treated with PBS (black dots, N = 13), LDN-212854 (red triangles, N = 13) or LDN-193189 (blue squares, N = 14). Data shown are mean ± SEM. Statistical significance was determined using an unpaired Student's *t* test.

Supplemental Figure 7

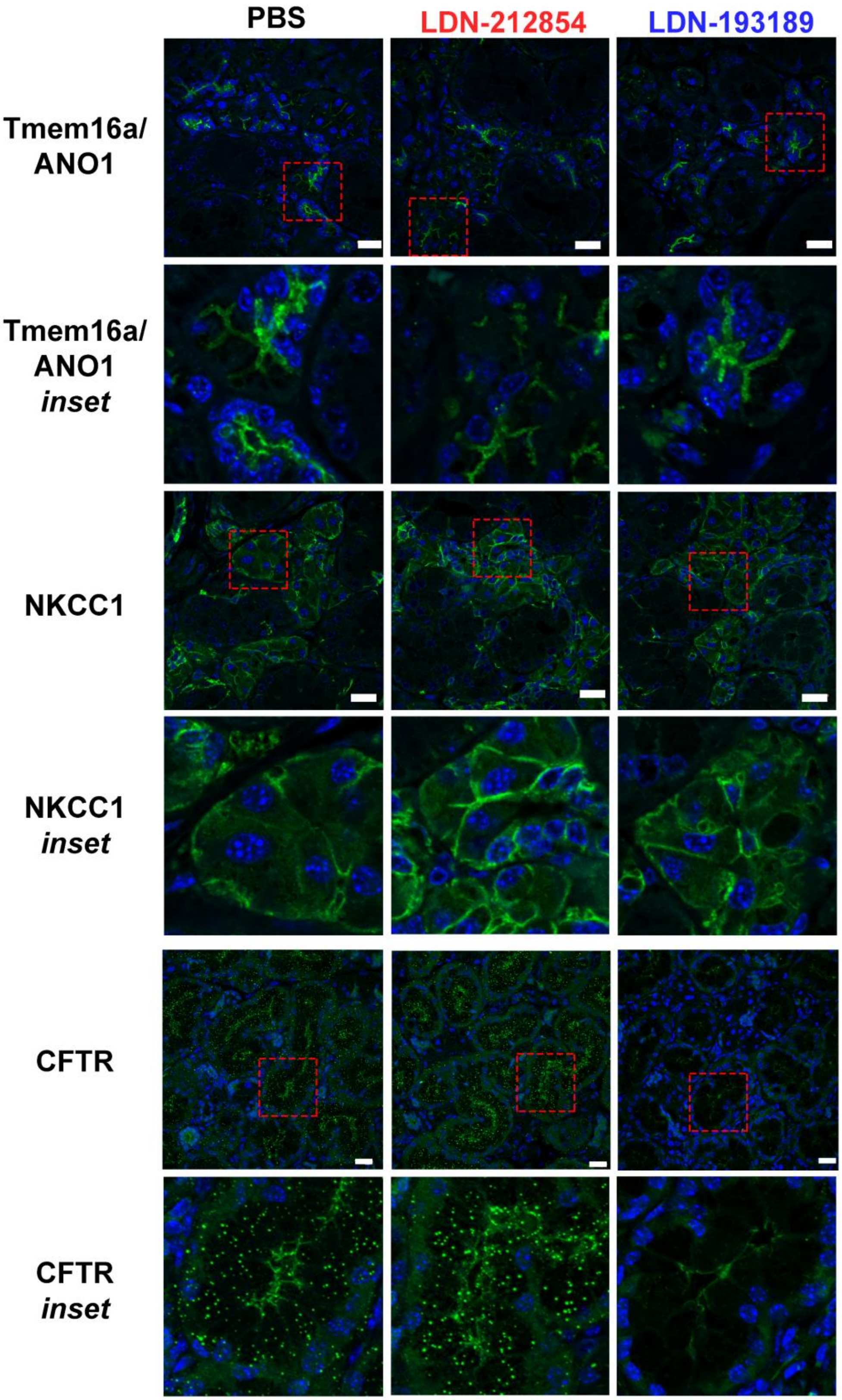

Supplemental Figure 7. Expression of TMEM16a/ANO1, NKCC1 and CFTR in submandibular glands of C57BL/6.NOD-*Aec1Aec2* mice

Representative confocal images of transmembrane member 16A (TMEM16a/ANO1), Na-K-Cl cotransporter 1 (NKCC1) and cystic fibrosis transmembrane conductance regulator (CFTR) expression in submandibular glands of C57BL/6.NOD-*Aec1Aec2* mice treated with PBS (left column), LDN-212854 (middle column), or LDN-193189 (right column). Row 1, 3 and 5: slides labeled with TMEM16a/ANO1, NKCC1and CFTR antibodies, respectively (40X magnification, white bar: 20μm). Row 2, 4 and 6: Inset for the marked area in row 1, 3 and 5, respectively (red-dashed box).
